# Robot Localization in Tunnels: Combining Discrete Features in a Pose Graph Framework

**DOI:** 10.3390/s22041390

**Published:** 2022-02-11

**Authors:** Teresa Seco, María T. Lázaro, Jesús Espelosín, Luis Montano, José L. Villarroel

**Affiliations:** 1 Instituto Tecnológico de Aragón, 50018 Zaragoza, Spain; mtlazaro@itainnova.es (M.T.L.); jespelosin@itainnova.es (J.E.); 2 Aragón Institute for Engineering Research (I3A), University of Zaragoza, 50009 Zaragoza, Spain; montano@unizar.es (L.M.); jlvilla@unizar.es (J.L.V.)

**Keywords:** localization, tunnel, pose-graph, inspection, RF propagation, robotics

## Abstract

Robot localization inside tunnels is a challenging task due to the special conditions of these environments. The GPS-denied nature of these scenarios, coupled with the low visibility, slippery and irregular surfaces, and lack of distinguishable visual and structural features, make traditional robotics methods based on cameras, lasers, or wheel encoders unreliable. Fortunately, tunnels provide other types of valuable information that can be used for localization purposes. On the one hand, radio frequency signal propagation in these types of scenarios shows a predictable periodic structure (periodic fadings) under certain settings, and on the other hand, tunnels present structural characteristics (e.g., galleries, emergency shelters) that must comply with safety regulations. The solution presented in this paper consists of detecting both types of features to be introduced as discrete sources of information in an alternative graph-based localization approach. The results obtained from experiments conducted in a real tunnel demonstrate the validity and suitability of the proposed system for inspection applications.

## 1. Introduction

Long road and railway tunnels (over 500 m) are important structures that facilitate communication and play a decisive role in the functioning and development of regional economies. Such infrastructure requires periodic inspections, repairs, surveillance, and sometimes rescue missions. The challenging conditions of these types of scenarios (i.e., darkness, dust, fluids, and toxic substances) make these tasks unfriendly and even risky for humans. These situations, combined with the continuous advances in robotic technologies, make robots the most suitable devices for executing these tasks.

For a robot to autonomously perform these tasks, it is essential to obtain an accurate localization, not only for the decision-making process but also to unequivocally locate possible defects detected during inspection works. However, some specific characteristics of these environments and their GPS-denied nature limit the type of sensors that can be used to acquire useful information for localization. Moreover, tunnel dimensions (with more length than width) and smoothness produce continuous growth in the longitudinal localization uncertainty that cannot easily be reduced.

The most common algorithms for indoor localization are those based on visual odometry (VO) using cameras whose core pipelines consist of the extraction and matching of features and LiDAR odometry (LO), which estimates the displacement of the vehicle by scan matching consecutive scans. These techniques can also be used within a simultaneous localization and mapping (SLAM) context to reduce localization uncertainty through loop closing.

However, a lack of visual features and sometimes darkness limit the use of vision sensors. Additionally, tunnel walls are uniform in long sections. Therefore, although laser sensors can be used to localize the robot in the cross-section, they do not provide useful localization information in the longitudinal dimension. Furthermore, odometers tend to suffer cumulative errors. Moreover, flying robots (e.g., quadrotors) lack odometers. The aforementioned issues make tunnel environments challenging for localizing purposes.

In recent years, promising technologies for indoor localization relying on the use of radio frequency (RF) signals have emerged as alternative methods. From the perspective of RF signal propagation, tunnel-like environments differ from regular indoor and outdoor scenarios. On the one hand, if the wavelength of the operating frequency is much smaller than the tunnel cross-section, the tunnel behaves as an oversized waveguide extending the communication range. On the other hand, the RF signal suffers from strong attenuations known as fadings. However, the occurrence of such fadings can be controlled. In [1], the authors developed an extensive analysis to determine the most adequate transceiver-receiver configurations (i.e., position in the tunnel cross-section and operating frequency) to obtain periodic fadings. Reference [2] drafted the first proposal to take advantage of the useful periodic nature of fadings to develop an RF-based discrete localization method. This characteristic was also exploited in the localization method described in [3]. In contrast to the use of ultra-wideband (UWB) or other RF localization techniques (e.g., based on access points), the generation and detection of periodic fadings do not require any previous infrastructure installation along the tunnel, which is not always possible.

By considering the aforementioned studies and recent advances in the field of graph SLAM, our previous work [4] addressed the robot localization problem in tunnels as an online pose graph localization problem for which we originally introduced the results of our RF signal minima detection method into a graph optimization framework by taking advantage of the periodic nature of RF signals within tunnels. Although the results were very promising, the distance between fadings is usually large (i.e., hundreds of meters), which causes the error to grow too much between detections.

Accidents can be very serious when they occur in tunnels. Due to the confined environment of tunnels, accidents—particularly those involving fires—can have dramatic consequences. As a result of tragic tunnel accidents in the European Union between 1999 and 2001, the European Commission developed a Directive [5] aimed at ensuring a minimum level of safety in road tunnels within the trans-European network. A total of 515 road tunnels over 500 m in length were identified. The total length of these tunnels is more than 800 km.

**Figure 1:**
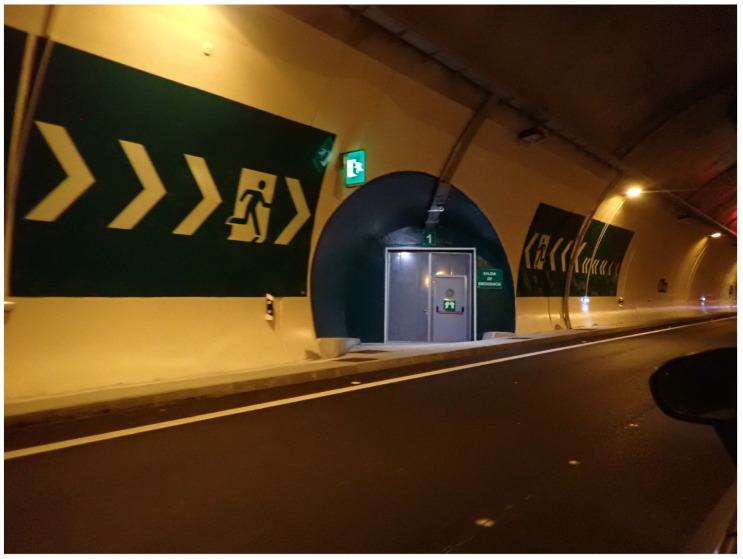
Example of emergency gallery in a road tunnel. Monrepos tunnel, Spain.

To be compliant with this directive, tunnels must integrate a set of structural elements to improve safety. Some examples from the European Directive are:

- Emergency exits (e.g., direct exits) from the tunnel to the outside, cross-connections between tunnel tubes, exits to emergency galleries, and shelters with an escape route separated from the tunnel tube (Figure 1). The distance between two emergency exits shall not exceed 500 m.
- In twin-tube tunnels where the tubes are at the same (or nearly the same) level, cross-connections suitable for the use of emergency services shall be provided at least every 1500 m.
- Emergency stations shall be provided near the portals and inside at intervals that shall not exceed 150 m for new tunnels.

These tunnel safety regulations provide a set of relevant structural characteristics that can be used for discrete localization inside tunnels. As can be extracted from the Directive, many of them are pseudo-periodic along the longitudinal dimension.

In view of the latter, we propose the complementary use of structural characteristics originating from the safety regulations to increase localization accuracy using only fadings. Thus, the alternative localization approach proposed in this paper builds on our aforementioned work [4], which is further extended with important new contributions that can be summarized as follows:

- First, two methods to identify tunnel galleries from onboard LiDAR information as relevant places for a global robot localization have been implemented and validated with real data.
- A strategy to introduce the results provided by a gallery detector into a pose graph has been developed.
- The improvement of localization along the tunnel using several discrete sources of information (fadings and galleries) is also demonstrated with experiments in a real-world scenario.
- Lastly, an exhaustive performance evaluation has been achieved to analyze the accuracy of the graph under different situations.

**Figure 2:**
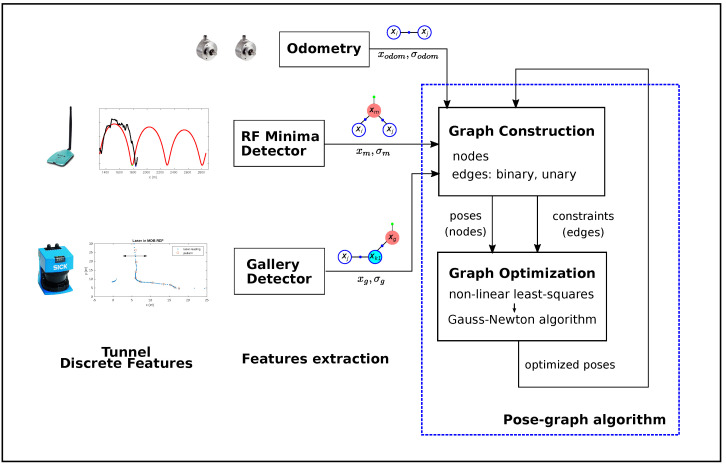
Overall schema of the graph-based localization approach.

Our approach consists of identifying discrete features from RF signals (minima) and structural characteristics (galleries) during the displacement of the robot along the tunnel. The resultant absolute positions provided by these detection methods are introduced as constraints in the pose graph together with the odometry measurements. Each time new information is incorporated into the graph, it is optimized and the position of the robot is corrected, which allows it to locate the main characteristics under inspection more accurately. Figure 2 shows an overall diagram of the proposed method.

The main advantages of adopting a graph-based representation are twofold: it allows for easier incorporation of delayed measurements into the estimation process and reverting from wrong decisions (e.g., the integration of incorrect measurements). Moreover, the use of different sources of information allows the resetting of cumulative localization error not only in the area of periodic fadings but also each time a gallery is detected.

The principal novelties of our method lie precisely in these advantages. Although graphs are used in other contexts, such as multi-sensor SLAM, it is not very common to find works where the graph is modified with past information. The application of the graph approach to tunnel-like environments is also not very widespread. Exploiting the RF periodic structure in those scenarios for localization constitutes another novelty of our proposed method.

The paper is structured as follows. The next section describes related work on the main technologies that our proposal is based on. Section 3 presents the real scenario and the robotic system used during the experiments. The challenges of the longitudinal localization in tunnels and how to face them are described in Section 4. Section 5 presents a detailed description of the methods used to detect discrete features (i.e., galleries and RF signal minima), while the graph-based localization approach and the mechanisms to introduce the detection results into the graph are presented in Section 6. Then, Section 7 discusses the experimental results obtained in a real-world scenario. Finally, the conclusions are summarized in Section 8.

## 2. Related Work

### 2.1. Place Recognition

Place recognition addresses the problem of determining the location of a sensor (possibly mounted on a mobile robot) by identifying some characteristics of the sensor reading and comparing it with a database or topological map. This has been widely used in SLAM to improve the accuracy of robot localization during loop closing. Place recognition has been an important line of research in the computer vision community (see [6]). This problem has primarily been tackled by extracting a set of locally-invariant features (e.g., SIFT or SURF points) from images. More recently, this problem has been approached by so-called Convolutional Neural Networks (CNN), or deep learning. However, as previously stated, the lack of visual features or darkness in tunnels can make these (in some cases mature) techniques fail.

The use of relevant structural characteristics for localization purposes in tunnel-like environments was explored in [7]. The authors presented a global localization system for ground inspection robots in sewer networks. This system takes advantage of the mandatory existence of manholes with a particular shape at known positions by identifying them (using machine learning techniques) and resetting the localization error. To achieve this, the authors used depth images provided by a camera placed on top of a robot and pointed toward the ceiling. Although their solution provided very good results, the use of cameras during the manhole detection process could be problematic in larger environments, as will be discussed in the following sections.

To overcome the illumination problem in the tunnel context, laser rangefinder sensors are of great importance. In this case, the sensor output is a point cloud that can be 2D or 3D. Place recognition can be performed in a similar manner as in vision (i.e., extracting relevant characteristics or keypoints from the point cloud). One possibility involves extracting the geometric characteristics (e.g., linearity, flatness, anisotropy) of objects present in the environment from the point cloud by following, for example, the method proposed in [8,9] to perform classification. Then, the relative position of objects determines the place. Another possibility involves the extraction of segments from the point cloud—as per [10]—for mapping and localization in the context of an urban environment based on 3D point clouds. The segments obtained allow the labeling of the scene to discriminate between vehicles and buildings, adding semantic information to the map. The referenced methods aim to reduce the uncertainty of localization by closing loops in which previously seen places are recognized by their appearance, without the need for a pre-built map. In [11,12], localization was performed based on 2D topological-semantic maps by recognizing semantic characteristics (e.g., crossings, forks, or corners) reflected in an existing topological map. This type of technique is adapted to 2D-structured underground environments such as mines.

It is also possible to extract a set of keypoints from the point cloud to describe a scene similarly to SIFT and SURF points extraction in vision. This was the approach followed in [13] for 2D point clouds, which were subsequently extended to 3D [14]. The authors proposed techniques for extracting relevant points from a point cloud and then used them in a database search or global map to identify the place.

In the present work, we must recognize two kinds of relevant features: lateral emergency galleries and RF signal fadings (virtual places). We propose two techniques for gallery detection: the first is based on keypoints that form a pattern identifying the gallery, the second is based on the extraction of straight regression lines from the scans fitting a generic gallery, which must satisfy certain restrictions. The identification of fadings is accomplished by an ad hoc technique based on the knowledge of the signal model.

### 2.2. Fundamentals of Electromagnetic Propagation in Tunnels; the Fadings

Previous works [2,15,16] have demonstrated that wireless propagation in tunnels differs from regular indoor and outdoor scenarios. For sufficiently high operating frequencies with free space wavelength much smaller than the tunnel cross-section dimensions, tunnels behave as hollow dielectric waveguides. If an emitting antenna is placed inside a long tunnel, the spherical radiated wavefronts will be multiply scattered by the surrounding walls. The superposition of all these scattered waves is itself a wave that propagates in one dimension—along the tunnel length—with a quasi-standing wave pattern in the transversal dimension. This allows an extension of the communication range but affects the signal with the appearance of strong fadings.

There are many different possible transversal standing wave patterns for a given tunnel shape. Each one is called a *mode* and has its own wavelength that is close to—but different from—the free space one, and with its own decay rate (see [17] for a detailed explanation).

The electromagnetic field radiated from the antenna starts propagating along the tunnel and is distributed via many of the possible propagating modes supported by this waveguide. Depending on the distance from the transmitter, two regions can be distinguished in the signal due to the different attenuation rates of the propagation modes. In the *near sector*, all of the propagation modes are present, which provokes rapid fluctuations in the signal (*fast-fadings*). After a sufficiently long travel distance, the higher-order modes (that have a higher attenuation rate) are mitigated and the lowest modes survive, giving rise to the so-called *far sector*, where the *slow-fadings* dominate [18]. Such fadings are caused by the pairwise interaction between the propagating modes. Therefore, the higher the number of modes, the more complex the fading structure. On the transmitter side, the position of the antenna makes it possible to maximize or minimize the power coupled to a given mode, thereby favoring the interaction between certain modes and facilitating the production of a specific fading structure. Therefore, the keypoint consists of attempting to promote the interaction of just two modes to obtain strictly periodic fadings.

**Figure 3:**
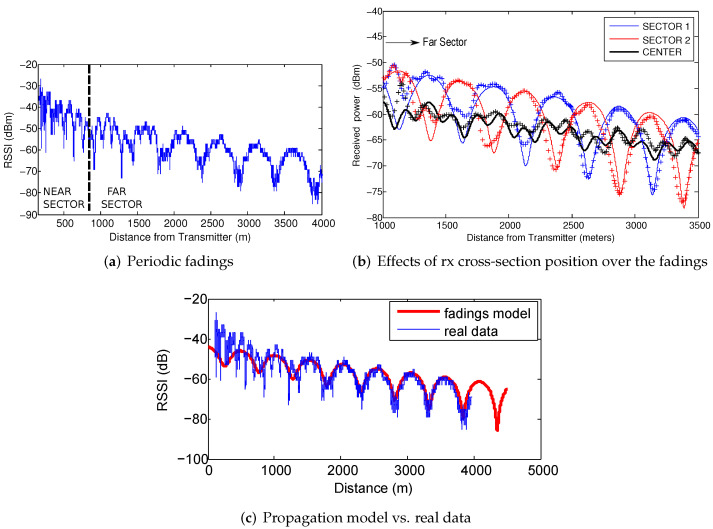
(**a**) Measured received power at 2.4 GHz inside the Somport tunnel, from [1]. The transmitter remained fixed and the receiver was displaced 4 km from the transmitter. In (**b**), the same experiment was repeated for three different receiver cross-section positions: left half (sector 1), center, and right half (sector 2). (**c**) shows the remarkable similarity between the propagation model and the experimental data. The red line represents the modal theory simulations, while the blue line represents the experimental results.

In [1], the authors presented an extensive analysis of the fadings structure in straight tunnels. These studies demonstrated that, given the tunnel dimensions and the selection of a proper transmitter-receiver setup, the dominant modes are the first three modes (i.e., the ones that survive in the far sector since their attenuation constant is low enough to ensure coverage along several kilometers inside the tunnel). By placing the transmitter antenna close to a tunnel wall, it is possible to maximize the power coupled to the first and second modes while minimizing the excitation of the third one. On the receiving side, this produces a strictly periodic fading structure. The superposition of the first and second propagation modes (called $EH_{11}^{z}$ and $EH_{21}^{z}$, respectively) creates a periodic fading structure (Figure 3a). In the very center of the tunnel, there is no contribution from the second mode, and the third mode ($EH_{31}^{z}$)—with lower energy—becomes observable, thereby creating another fading structure with a different period. However, the received power associated with the fadings maxima is lower compared to the previous fading structure. This situation is illustrated in Figure 3b, which shows the data collected by having one antenna in each half of the tunnel and another located in the center. Evidently, there is a spatial phase difference of 180 degrees between both halves of the tunnel (i.e., a maximum of one fading matches the minimum of the other) caused by the transversal structure of the second mode. It is important to highlight that we refer to large-scale fadings in a spatial domain, which is a standing wave pattern that can be obtained in tunnels under certain configurations, in contrast to the well-known small-scale fadings, understood as temporal variations in a channel.

Lastly, in the presented studies, the authors adopted the modal theory approach, which models the tunnel as a rectangular dielectric waveguide of dimensions *a*×*b* using the expressions for the electromagnetic field modes and the corresponding propagation constants obtained by [19] for rectangular hollow dielectric waveguides. As previously mentioned, each mode propagates with its own wavelength $\lambda_{mn}$ (close but not equal to the free space one), which can be written as:

(1) $$\lambda_{mn}=\frac{\lambda }{1-\frac{1}{2}{\left(\frac{m\lambda }{2a}\right)}^{2}-\frac{1}{2}{\left(\frac{n\lambda }{2b}\right)}^{2}}$$

where *m* represents the number of half-waves along the *y* axis, *n* the number of half-waves along the *z* axis and λ is the free space wavelength that depends on the free space velocity of the electromagnetic waves *c* and the operating frequency *f*:

(2) $$\lambda =\frac{c}{f}$$

If two modes with different wavelengths ($\lambda_{1}$ and $\lambda_{2}$) are present, the phase delay accumulated by each one will be different for a given travel distance *x*. The superposition of the modes will take place with different relative phases in different positions within the guide, producing constructive interference if both modes are in phase and destructive interference if the relative phase differs by an odd π multiple. This gives rise to a periodic fading structure of the RF power inside the waveguide. The period of this fading structure *D* is the distance, which creates a relative phase of 2π between the two considered modes. If $\lambda_{1}$ and $\lambda_{2}$ are the wavelengths of the two modes, then:

(3) $$D=\frac{\lambda_{1}\lambda_{2}}{|\lambda_{1}-\lambda_{2}|}$$

Using Equation (1), the fading period obtained is:

(4) $$D=\frac{8}{\frac{c}{f}\left|\frac{m_{2}^{2}-m_{1}^{2}}{a^{2}}+\frac{n_{2}^{2}-n_{1}^{2}}{b^{2}}\right|}$$

As can be deduced from Equation (4), the period of fadings only depends on the operating frequency and the dimensions of the tunnel. The total electromagnetic field, which represents the propagation model, will be the superposition of all the propagation modes (see [1] for a complete 3D fadings structure analysis in tunnels).

With this approximation, the obtained theoretical propagation model closely matches the experimental data. The similarity between both signals (Figure 3c) in the *far sector* is sufficient to make us consider them useful for localization purposes, using the propagation model as a position reference.

### 2.3. Graph-Based Localization

The SLAM problem is one of the fundamental challenges of robotics since it deals with the need to build a map of an unknown environment while simultaneously determining the robot localization within this map. In the literature, a large variety of solutions to this problem are available, which are usually classified into filtering and optimization (graph) approaches. The latter offers improved performance and the capability of incorporating information from the past, having memorized all data. Moreover, it facilitates the incorporation of relative and absolute measurements coming from different sources of information.

All of these advantages led to the emergence of new localization approaches that model the localization problem as a pose graph optimization. The pose graph encodes the robot poses during data acquisition as well as the spatial constraints between them. The former are modeled as nodes in a graph and the latter as edges between nodes. These constraints arise from sensor measurements.

In [20], the authors provided a positioning framework targeted for agricultural applications. They integrated several heterogeneous sensors into a pose graph in which the relative constraints between nodes were provided by wheel odometry and VO, while the global (so-called prior) information was provided by a low-cost GPS and an Inertial Measurement Unit (IMU). The proposed system also introduces further constraints exploiting the domain-specific patterns present in these environments. The effectiveness of incorporating prior information was also demonstrated in [21]. The presented solution relies on a graph-based formulation of the SLAM problem based on 3D range information and the use of aerial images as prior information. The latter was introduced to the graph as global constraints that contain absolute locations obtained by Monte Carlo localization on a map computed from aerial images. Similarly, Reference [22] also proposed the use of prior constraints to improve localization in industrial scenarios. In that study, prior information was provided by a CAD drawing that allowed the robot to estimate its current position with respect to the global reference frame of a floor plan. Another approach is presented in [23], where robot localization in water pipes is improved by incorporating acoustic signal information into a pose graph.

Furthermore, graph-based approaches provide an effective, flexible and robust solution against wrong measurements and outliers as proved in works such as [24,25], which allow it to recognize and recover from outliers during the optimization time.

In light of the aforementioned works, our approach consists of addressing the localization in tunnels as a pose graph optimization problem, incorporating the data provided by the detectors of the relevant discrete features.

## 3. The Canfranc Tunnel and Experiments Setup

The Somport road tunnel is a cross-border monotube tunnel between Spain and France through the central Pyrenees, situated at an altitude of 1100 m and with a length of 8608 m (two-thirds in Spanish territory and one-third in French territory). The tunnel runs parallel to the Canfranc railway tunnel, which is currently out of service and acts as the emergency gallery. Both tunnels are connected by 17 lateral galleries that serve the function of emergency exits for the road tunnel.

The Canfranc railway tunnel has a length of 7.7 km. The tunnel is straight but suffers a change in slope at approximately 4 km from the Spanish entrance. It has a horseshoe-shaped cross-section that is approximately 5 m high and 4.65 m wide. The tunnel has small emergency shelters every 25 m. The lateral galleries are more than 100 m long and of the same height as the tunnel. The galleries are numbered from 17 to 1, from Spain to France.

The experiments of the present work were conducted in the Canfranc tunnel.

An instrumented all-terrain vehicle was used as the mobile platform to simulate a service routine. It was equipped with two SICK DSF60 0.036 deg resolution encoders that provide the odometry information and a SICK LMS200 LiDAR intended for gallery detection.

**Figure 4:**
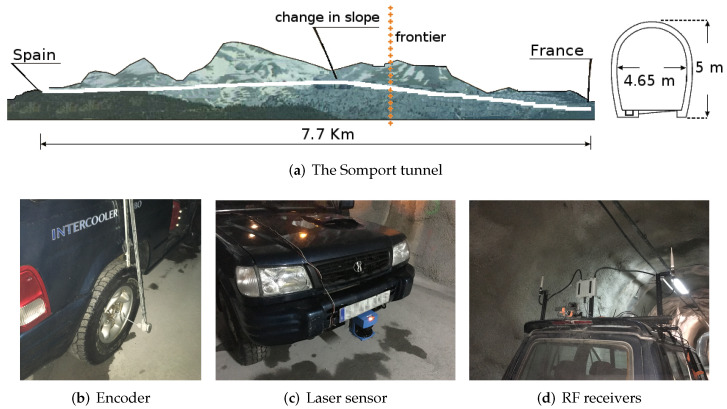
Experimental setup: Somport tunnel dimensions (**a**) and instrumented vehicle (**b**–**d**).

The platform was also equipped with two RF ALFA AWUS036NH receivers placed 2.25 m above the ground with the antennas spaced 1.40 m apart. The transmitter, a TPLINK TL-WN7200MD wireless adapter with Ralink chipset, was placed at approximately 850 m from the entrance of the tunnel, 3.50 m above the ground, and 1.50 m from the right wall. Using a 2.412 GHz working frequency under this receiver-transmitter setup, the expected fadings period is approximately 512 m. The RF data used to validate the proposed method are the received signal strength indicator (RSSI) values provided by the rightmost antenna. Figure 4 shows the experimental setup.

## 4. Localization: Problem Formulation

**Figure 5:**
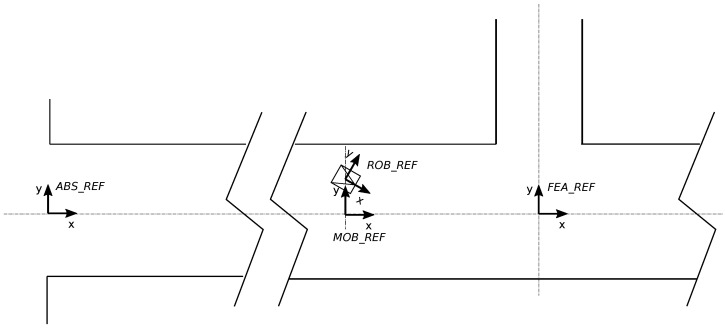
Involved reference frames in the robot localization problem.

The reference systems involved in the localization procedure are shown in Figure 5 and defined as follows:

ABS_REF (A): Global or absolute reference frame of the tunnel. It is located at the middle of the tunnel entrance (i.e., over the tunnel axis).
ROB_REF (R): Local reference frame of the mobile robot. It is located at a point on the robot chassis.
MOB_REF (M): Sliding reference over the tunnel axis. It has the same orientation as the tunnel axis and the same absolute *x* position as the ROB_REF.
FEA_REF_i (F): The relevant i-th feature (gallery, fading, or other) is expressed in this reference. It is located at the tunnel axis in the feature location.

Road and railway tunnels may have curves. However, modern tunnels present curves with a large bending radius, which allows us to consider them to be rectified as if they were nearly straight. Thus, we maintain as *x* coordinate the actual distance on the tunnel axis line (straight or curved) in the absolute reference *A* while *y* and θ coordinates are represented in the mobile reference *M* (see Figure 5), centered in the instantaneous axis of the robot at every moment. In this manner, since the mission is to navigate the robot to explore or inspect a tunnel, it is not necessary to compute its curvature with respect to the initial location. To achieve this, the absolute $x_{r}$ robot coordinate will be recomputed by matching the absolute location $x_{g_{i}}$ of the detected gallery and its corresponding $x_{g_{i}}^{A}$ in the prior tunnel map.

According to this, in the robot location $x_{r}=\left(x_{r},y_{r},\theta_{r}\right)$, $x_{r}$ will be computed in the *A* reference and the $y_{r}$ and $\theta_{r}$ coordinates will be computed in the mobile *M* reference. Then, we divide the localization problem into two subproblems: transverse localization and longitudinal localization. The transverse localization consists of calculating $y_{r}$ and $\theta_{r}$ in the *M* reference while the longitudinal localization involves calculating $x_{r}$ in the *A* reference along the central axis of the tunnel.

Transverse localization can be conducted with precision using a laser range sensor, as will be presented in Section 4.1. However, longitudinal localization is more problematic. This is introduced in Section 4.2 and developed in the following sections.

Hereafter, in order not to overload the nomenclature, the reference system will only be indicated in the case of the magnitude being referred to a system other than ABS_REF. The only exception will be when referring to reference positions provided by maps (e.g., the gallery position provided by the map $x_{g}^{A}$).

### 4.1. Transverse Localization in a Tunnel

To establish the transverse location of the robot, we use a flat beam laser rangefinder (see Section 3). Since we claim that transverse localization is not a problem, we use one of many available techniques. Firstly, a filtering of the laser scan points is needed. The terrain in a tunnel can be uneven, which provokes robot vibrations affecting the onboard laser rangefinder. As a result, some points in front of the robot belonging to the floor can appear. These points are filtered to only process those belonging to the walls.

**Figure 6:**
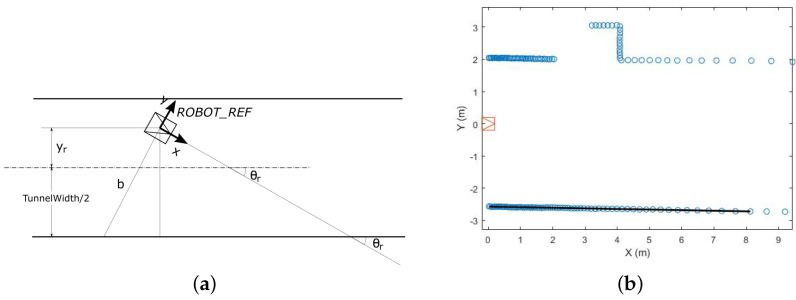
Robot transverse localization inside a tunnel. (**a**) Localization parameters referred to the right wall. (**b**) Regression straight line of the right wall.

In each control period, the transverse robot localization is computed as follows (see Figure 6). A regression straight line is computed from the scan points corresponding to the right and left walls. The line with the smallest fitting error is then selected. Let y=ax+b, the equation of this line in the ROB_REF. Then, the robot orientation $\theta_{r}$ is computed as: $\theta_{r}=-\arctan\left(a\right)$, being $\theta_{r}=0$ the orientation parallel to the wall. From the bias *b* of the line associated with the wall and the orientation $\theta_{r}$, the $y_{r}$ coordinate is computed with respect to the tunnel central axis: $y_{r}=b\cos\theta_{r}-TunnelWidth/2$.

The standard deviations $\sigma_{y}$ and $\sigma_{\theta }$ are computed from the mean squared error of the regression line. Let δ be the 95% prediction interval resulting from the regression computation. The standard deviation of *b* can be approached by $\sigma_{b}=\delta /2$ and the standard deviation of *a* by $\sigma_{a}=2\delta /d$, where *d* is the length of the regression line. Then:

$$\sigma_{\theta }=\frac{\sigma_{a}}{1+a^{2}}$$

$$\sigma_{y}=\sqrt{cos^{2}\left(\theta_{r}\right)\sigma_{b}^{2}+b^{2}sin^{2}\left(\theta_{r}\right)\sigma_{\theta }^{2}}$$

With this simple technique, the following uncertainties in robot location are obtained:
Standard Deviation% of Samplings$\sigma_{y}<0.02$ m96%$\sigma_{y}<0.03$ m98%$\sigma_{\theta }<1^{\circ }$96%$\sigma_{\theta }<2^{\circ }$98%

These values have been calculated in a robot trajectory of 4 km long inside the Canfranc tunnel. As can be seen, uncertainty in the transverse location can be neglected with respect to uncertainty in the longitudinal location. This will be revealed in the following sections.

### 4.2. Longitudinal Localization in a Tunnel

Identifying a robot’s position in the cross-section of tunnel-like environments could be achieved using traditional techniques, such as the one presented in Section 4.1. However, localization along the longitudinal axis represents a challenge.

Due to the absence of satellite signal in underground scenarios, outdoor methods based on GPS sensors cannot be used. Additionally, the darkness and lack of distinguishable features make the most common techniques for indoor localization—based on cameras or laser sensors—perform erratically. The work in [26] presented an autonomous platform for exploration and navigation in mines, where localization is based on the detection and matching of natural landmarks over a 2D survey map using a laser sensor. However, these methods are inefficient in monotonic geometry scenarios with the absence of landmarks, as shown in [27]. Recent alternatives based on visual SLAM techniques ([28,29]) rely on the extraction of visual features using cameras to provide accurate localization. These methods are highly dependent on proper illumination, which is usually poor in these types of environments. Moreover, they do not perform well in low-textured scenarios where the feature extraction process tends to be unstable.

The aforementioned issues have been stated in several works (e.g., [12,30]) using LiDAR-based systems. This problem has also been studied and formalized in [31], where a tunnel was considered as a geometrically degenerated case. The authors proposed fusing LiDAR sensor information with a UWB ranging system to eliminate degeneration. However, this solution involved the installation of a set of UWB anchors in the tunnel to act as RF landmarks. That is, the tunnel was modified to introduce artificial features for localization only. A similar solution was proposed in [32], which presented a robotic platform capable of autonomous tunnel inspection that was developed under the European Union-funded ROBO-SPECT research project. The authors stated that robot localization in underground spaces and on long linear paths is a challenging task. Again, artificial physical landmarks were also placed within the tunnel infrastructure to solve this problem.

Other localization methods rely on wheel odometers. However, besides suffering from cumulative errors, these are more unreliable than usual due to uneven surfaces and the presence of fluids being very common in tunnel environments.

To overcome all of the aforementioned difficulties, we propose an alternative method that combines different sources of information provided by the environment in a pose graph framework. For longitudinal robot localization, three types of information are integrated:

- The odometry or a rangefinder scan matching continuous localization. If the tunnel walls do not have sufficient roughness (i.e., flat walls), scan matching cannot provide information to localize the robot in the longitudinal coordinate. Therefore, in this work we consider odometry as the only available continuous source of information to make the results more generalizable.
- Geometric relevant features found along the tunnel. In this case, they correspond to lateral galleries, which are identifiable as discrete features. The absolute locations of these features are represented in a prior map. Two methods are developed for their identification and localization.
- Minima in RF communication signal, whose positions along the tunnel are known from a propagation model.

During motion, continuous localization $x_{r}$ will be maintained for the robot, computing $x_{r}$ from the odometry and computing $y_{r}$ and $\theta_{r}$ coordinates as explained in Section 4.1. The coordinate $x_{r}$ will be updated every time a signal minimum or geometric feature (a gallery in this case) will be identified. The next section is devoted to the development of methods for identifying and localizing both types of features.

## 5. Discrete Features Detection: Galleries and Fadings

We propose the use of sparsely distributed features present in tunnel environments to improve robot localization. In the present work, two sources of information are selected to be introduced in the pose graph: the absolute position corresponding to the RF signal minima and the absolute position of the galleries present in the tunnel. This section describes the detection methods of both types of features and their outputs, which will be subsequently added to the localization process.

### 5.1. Emergency Gallery Detection

We assume that the robot heading and transverse position are calculated with sufficient precision (Section 4.1) from sensor readings. Since both are known, the problem is addressed as 1D.

Two techniques are presented for emergency gallery recognition. The first (*Pattern*) is based on scan-pattern matching. The main difference with respect to traditional scan matching processes is that it does not attempt to match single scans, but rather matches scans with a gallery pattern (set of keypoints) in a prior metric-topological map. This technique is used when there is detailed prior knowledge of the tunnel galleries. The second technique (*Generic*) is developed to recognize generic lateral galleries without previous knowledge of their specific geometry. *Generic* can be used when the gallery is explored for the first time to obtain a geometric map for later use. Once the tunnel has been explored, *Pattern* can exploit the prior map to obtain a more precise localization while the robot is navigating to accomplish a mission inside the tunnel. *Generic* could be also used as a complementary to *Pattern* to increase the robustness of the recognition. Both techniques are described in the following subsections.

#### 5.1.1. Pattern Matching Gallery Detection

The following points summarize the steps of this gallery detection approach:

1. A unique pattern is extracted from the whole scanned tunnel that represents each of the galleries. The pattern is composed of a set of relevant points (keypoints) extracted from the metric-topological map of the tunnel: (${\left\{p_{j}\right\}}_{g}^{F},\;j\in \left[1\ldots m\right],\;p_{j}=\left(p_{x},p_{y}\right)$) in the FEA_REF feature reference.
2. All information regarding the galleries is saved in a database that contains the following for each gallery $g_{i}$: the pattern points (${\left\{p_{j}\right\}}_{g_{i}}^{F})$), the global localization of the gallery ($x_{g_{i}}^{A}$), and a sequential unique identification along the tunnel ($id_{g}$). This information is available in advance.
3. A scan-pattern matching process takes place to identify the gallery that has been traversed by the robot in real-time.
4. The gallery is detected from several robot positions as it passes by (before and after the reference position of the gallery defined by the pattern). The relative distance between each of the robot positions and the gallery, and the uncertainty of the detection are provided during the matching process.
5. Once a gallery is detected and knowing the current localization and movement direction of the robot, the next pattern is extracted from the database and the scan-pattern matching process starts again.

As previously stated, the pattern must unequivocally identify each gallery. It consists of a set of *m* relevant points ${\left\{\left(p_{x_{1}},p_{y_{1}}\right),\ldots ,\left(p_{x_{m}},p_{y_{m}}\right)\right\}}^{F}$ extracted from the geometric map, which represents the contour of the gallery.

**Figure 7:**
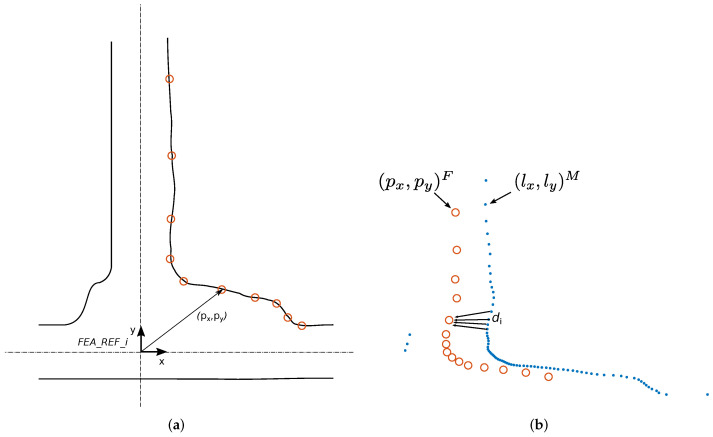
Pattern matching gallery detection (gallery 17 of the Canfranc tunnel). (**a**) Pattern extraction. The origin of the pattern reference system FEA_REF_i corresponds to the intersection of two lines: the axis of the tunnel and the axis of the gallery (in the present example). (**b**) NNS method applied between the laser and pattern points.

The global localization of a gallery ($x_{g_{i}}^{A}$) usually corresponds to a relevant feature of the gallery (e.g., the right or left corner or the axis). This question must be determined in the topographic work of the tunnel. In the present work, the localization of a gallery corresponds (without the loss of generality) to the intersection of the gallery axis and tunnel axis. The origin of the FEA_REF refers to this point (Figure 7a) and thus to this known gallery position ($x_{g_{i}}^{A}$). The $\left(p_{x},p_{y}\right)$ values for each point of the pattern are calculated with respect to this reference system.

For scan-pattern matching to work correctly, the laser data referenced with respect to the ROB_REF frame must be aligned with the pattern, which is defined with respect to the FEA_REF frame. This is performed by converting the laser points (${\mathrm{lp}}^{R}$) to the MOB_REF frame (${\mathrm{lp}}^{M}$) by means of applying a rotation and translation corresponding to the robot orientation $\theta_{r}$ and $y_{r}$ position, which are calculated using the method described in Section 4.1. In this manner, both the pattern scan points representing the gallery in the metric-topological map and the ones corresponding to the gallery and detected during motion are aligned in MOB_REF. Thus, there is only a need to compute the relative displacement in the matching process.

Once the laser points and the pattern are aligned, the matching process takes place.

**Metrics definition**

The first step in scan-pattern matching is the definition of a metric to measure the distance between the pattern and the scan reading. For this work, we have selected the nearest point-to-point metric. However, one can easily use any other type of metric (e.g., point-to-line metrics).

The nearest neighbor search (NNS) method is widely used for pattern recognition applications. The NNS problem in multiple dimensions is stated as follows: given a set *S* of *n* points and a novel query point *q* in a d-dimensional space, find the closest point in the set *S* to *q*. In this particular case, it consists of finding the nearest point from the laser data ${\left\{\left(l_{x_{1}},l_{y_{1}}\right),\ldots ,\left(l_{x_{n}},l_{y_{n}}\right)\right\}}^{M}$ to each point of the pattern ${\left\{\left(p_{x_{1}},p_{y_{1}}\right),\ldots ,\left(p_{x_{m}},p_{y_{m}}\right)\right\}}^{F}$ computing the corresponding distances. This method provides a distance vector $\left(d_{1},d_{2},\ldots ,d_{m}\right)$ of size *m*, with *m* being the number of points of the pattern (Figure 7b). The position error between the pattern and the scan reading is calculated as the mean quadratic error of the distance vector using Equation (5).

(5) $$err_{x}=\sqrt{\frac{1}{m}\sum_{i=1}^{m}d_{i}^{2}}$$

For each iteration, the matching process between the laser data and the gallery pattern using the NNS algorithm is applied for a range of [−r,+r] meters around the reference position of the pattern (FEA_REF). The distance in that range for which the matching process provides the least position error is obtained. This distance will correspond to the relative position between the robot and the reference position from where the pattern is captured (i.e., the relative distance between the robot position and the gallery ($d_{rg}$)). A gallery is considered detected if the corresponding position error is lower than a defined threshold (th).

Once the gallery is considered detected using the position error criteria, the next step is to unequivocally identify the gallery the robot is passing by, avoiding false positives. For this purpose, knowing the estimated position of the robot $x_{r}$ at this time and the absolute position of the gallery provided by the metric-topological map $x_{g_{i}}^{A}$, both values should be close enough to consider that gallery *i* has been identified:

(6) $$x_{r}\in \left[\left(x_{g_{i}}^{A}-H\right),\left(x_{g_{i}}^{A}+H\right)\right]$$

where *H* is a safety margin distance and it is proportional to the odometry error accumulated since the last identified feature.

It should be noted that the starting position of the robot is known, and therefore, the data corresponding to the first gallery to be identified (pattern, global position, and identification) can be extracted from the database. Once the gallery is detected, and knowing the direction of movement, the next gallery information is loaded into the matching process for subsequent identification. This strategy avoids attempting to match the current laser readings with all available patterns, improving the efficiency of the detection process.

In summary, the results of the emergency gallery detection algorithm at each timestamp are, on the one hand, the relative distance ($d_{rg}$) between the current position of the robot ($x_{r}$) and the gallery position (defined by the pattern reference system), and on the other hand, the gallery detection uncertainty ($\sigma_{rg}$). A gallery is considered detected from the robot if the uncertainty value is below a certain threshold (th). The absolute real position of each gallery is also known from the map of the tunnel ($x_{g_{i}}^{A}$). It is worth noting that each gallery will be detected from different robot positions in the defined search area around the pattern reference position [−r,+r].

#### 5.1.2. Generic Gallery Detection

The technique described above can only be used when a map has been constructed. However, no map is available the first time a new scenario is explored, and therefore, a technique not based on previous dense geometric maps is required. It should be enough to have the location of a representative point of each gallery ($x_{g_{i}}^{A}$) and a sequential identification along the tunnel ($id_{g}$) included in a topological map.

This method can recognize right and left lateral galleries along a principal long tunnel. The galleries can be of any shape, length, width, and inclination with respect to the principal corridor. To make this method generic for the identification of galleries in any tunnel, only a set of ranges for these generic parameters defining the features (lateral galleries in this case) from prior knowledge of the tunnel must be provided to the algorithm: width of the tunnel and galleries, number of supporting points of a gallery and distance between galleries. The galleries are recognized by using angular and distance constraints between the lines delimiting these features, obtained from a regression technique (as explained in Section 4.1). Therefore, it is not necessary to maintain a dense geometric map of the galleries. In this case, only the location of the representative point of each gallery in the global reference ($x_{g_{i}}^{A}$) is required.

**Figure 8:**
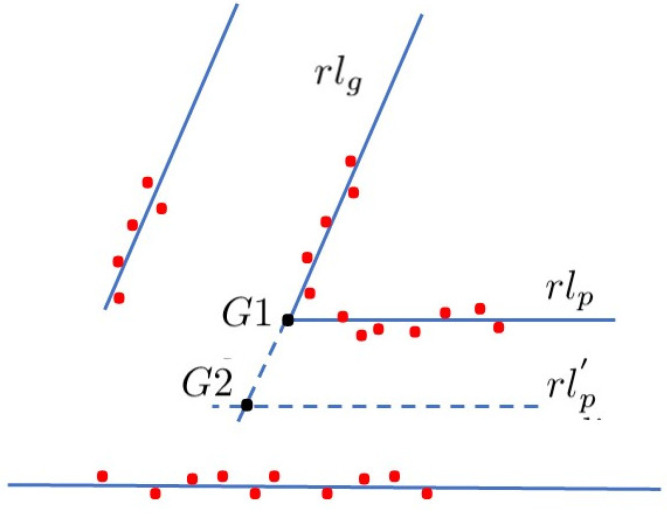
Computation of the representative point *G* of a generic gallery. The point G1 or G2 having the lower location uncertainty is chosen as the representative point of the gallery.

The method evolves as follows:

1. A topological map including the ordered global localization of the galleries ($\left[x_{g_{1}}^{A},\ldots ,x_{g_{i}}^{A},\ldots ,x_{g_{n}}^{A}\right]$) is available. No geometric or scan information are required.
2. The robot autonomously moves or is driven along the main corridor, detecting and recognizing the correspondent gallery in the prior topological map using the aforementioned geometric constraints.
3. When a potential gallery $g_{i}$ is detected from the scans taken from the successive robot positions, a recognition process is launched. In this process, the geometric constraints defining a generic gallery—invariant to its relative robot pose—are tested from several sequential scans taken while the robot is moving. If the constraints are met, the gallery is validated. In this manner, other features in the tunnel (e.g., shelters, holes, or wall irregularities) are filtered out.
4. The localization of the validated gallery $g_{i}$ is computed. The global location of the gallery in the topological map is assigned to the representative point with lowest uncertainty, G1 or G2 in Figure 8. The new robot absolute coordinate $x_{r}$ is obtained from the corresponding G1 or G2 computed from the LIDAR sensor points when a gallery is identified. Finally, the complete robot location $x_{r}$ is computed by using the transverse localization method detailed in Section 4.1.
5. The number and localization of the next gallery $g_{i+1}$ is extracted from the topological map and the recognition process starts again.

As per the *Pattern* matching method, the laser data are expressed in the MOB_REF. Then, the scan points are segmented, computing the associated regression straight lines, which delimit the area around the tunnel gallery and constitute the main information for the gallery detector. To make the process robust, a minimum number of supporting points are needed to compute the lines and recognize a gallery. As the detected points change as the robot moves forward, the gallery is only validated when it is identified from several consecutive positions. False positives are this way avoided.

When a gallery is recognized, the robot localization uncertainty is computed from the intersection point G1 (see Figure 8) between the regression lines $rl_{g}:y=a_{g}x+b_{g}$ and $rl_{p}:y=a_{p}x+b_{p}$ of the corner, respectively. Another possibility involves computing the point G2, which is the intersection between a virtual line parallel to the opposite wall in the robot, which is computed in the robot reference *R*, $rl{{}^{\prime }}_{p}:y=a_{p}x$, being $a_{p}=tan\left(-\theta_{r}\right)$. The computation leading to the lowest uncertainty is used. The *x* coordinate of the intersection point in *R* is computed as $x_{g}=\frac{b_{p}-b_{g}}{a_{g}-a_{p}}$. Let $x_{g}^{A}$ be the *x* coordinate of the *G* point representative of the gallery location in the global reference *A* (ABS_REF, Figure 5) obtained from the topological map.

The coordinate $x_{r}$ of the robot in *A* can be computed as:

(7) $$x_{r}=x_{g}^{A}-\rho cos\left(\theta_{r}+\alpha \right)$$

where ρ and α are the distance and angle measured from the *G* point to the origin of the *R* reference, respectively, and $\theta_{r}$ is the robot orientation computed as explained in Section 4.1.

The variance of this estimation is obtained from the variance of the *x* coordinate of the *G* point and the variance of the robot orientation $\theta_{r}$. The variance $\sigma_{x_{g}}^{2}$ of *G* is computed from the variances of the parameters of both regression lines. Therefore, the variance $\sigma_{xr}^{2}$ is computed as:

(8) $$\sigma_{xr}^{2}=JCJ^{T}$$

being

$$J=J\left(x_{g}^{A},\theta_{r}\right)=\left(\frac{b_{g}}{{\left(k_{g}-k_{p}\right)}^{2}},\frac{-1}{a_{g}-a_{p}},\frac{b_{p}-b_{g}}{{\left(a_{g}-a_{p}\right)}^{2}},\frac{1}{a_{g}-a_{p}},\rho \sin\left(\theta_{r}+\alpha \right)\right)$$

$$C=diag(\sigma_{a_{g}}^{2},\sigma_{b_{g}}^{2},\sigma_{a_{p}}^{2},\sigma_{b_{p}}^{2},\sigma_{\theta_{r}}^{2})$$

### 5.2. RF Signal Fading Detection

**Figure 9:**
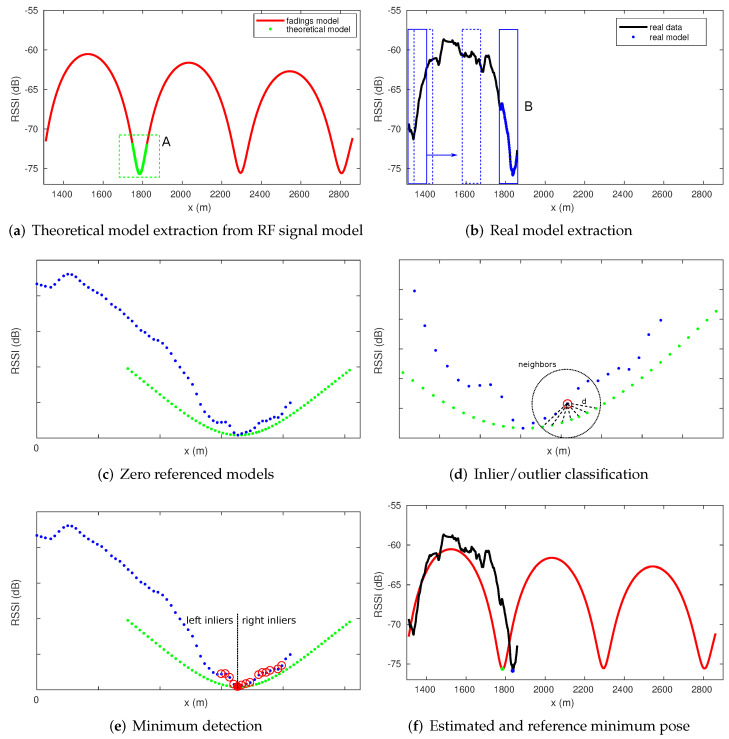
RF signal minima detection steps: (**a**) Theoretical model (green points inside dashed green square A) extracted from the RF signal model (red). (**b**) Real model generation during the displacement of the vehicle from the real RF signal. (**c**) Both models referenced the same system coordinates. (**d**) Point classification depending on the Mahalanobis distance between the real data and the closest neighbors from the theoretical model. (**e**) Minimum detection if the number and proportion of inliers satisfy the threshold. (**f**) Estimated position based on the odometry $x_{m}$ (blue point) and position reference from the RF map $x_{m_{i}}^{A}$ (green point) of the detected minimum.

The first step of the proposed method consists of extracting a discrete model representing the theoretical minimum model from the RF map. Using the propagation model described in [1], it is possible to determine the position of each valley along the tunnel, and the theoretical minimum model can then be obtained in advance. This model consists of a set of points (x,rs) where *x* represents the position and rs the theoretical RSSI value (Figure 9a). During the displacement of the vehicle, the algorithm attempts to match a discrete real model with the theoretical model. The real model is generated by accumulating points $\left(x_{t},rs_{t}\right)$ during a certain period of time (Figure 9b), where $x_{t}$ is the position estimated by the odometry and $rs_{t}$ corresponds to the actual RSSI value provided by an RF sensor.

The matching process is based on the calculation of the Mahalanobis distance between each real point from the real model and the closest neighbors from the theoretical model (Figure 9c,d). The points are classified as inliers or outliers depending on the resultant distance. If the number of inliers is greater than a certain threshold and the ratio between left and right inliers is balanced, we can conclude that a minimum has been found (Figure 9e). Information about the estimated position of the minimum $x_{m}$ together with its corresponding position in the map $x_{m_{i}}^{A}$ is available (Figure 9f). The RF minimum detection method is explained in detail in [4].

The resulting data are the estimated position provided by the odometry ($x_{r}=x_{m})$ and the position reference of the RF map ($x_{m_{i}}^{A}$), both of which correspond to a minimum of the RF signal. The uncertainty of the position reference ($\sigma_{m}$) is a measure of the RF signal model fidelity with respect to the ground truth. This value is initially estimated offline based on the absolute position of the transmitter in the tunnel. Subsequently, it is adjusted online by a practical approach after each trip, comparing the positions of the actual minima, provided by the localization approach, with the absolute positions of the minima of the RF signal model.

It is remarkable that the information provided by the virtual sensor corresponds to delayed measurements (i.e., the position of the minimum is detected at a timestamp (*T*) after its appearance (T−k)). This implies the incorporation of information referring to a past position in the estimation process. This can be managed through the use of a graph representation, having an impact on the current pose estimation after the optimization process. The strategy followed to add these measurements to the pose graph is explained in Section 6.1.

## 6. Multi-Sensor Graph-Based Localization

Inspired by the graph SLAM paradigm, our approach models the robot localization problem as a graph-based pose optimization problem. This approach represents the robot trajectory $x_{0:T}=\left\{x_{0},\ldots ,x_{T}\right\}$ using a graph in which nodes symbolize discrete robot positions $x_{t}$ at time step *t* while the edges impose position constraints on one or multiple nodes [33]. Hence, some nodes in the graph are related by *binary* edges encoding relative position constraints between two nodes $\left(x_{i},x_{j}\right)$ characterized by a mean $z_{ij}$ and an information matrix $\Omega_{ij}$. These relative measurements are typically obtained through odometry or scan matching. Furthermore, it is possible to incorporate global or prior information associated with only one robot position $x_{i}$ into the graph by means of *unary* edges characterized by the measurement $z_{i}$ with information matrix $\Omega_{i}$. These measurements typically come from sensors providing direct absolute information about the robot pose (e.g., GPS or IMU).

Let $x={\left(x_{0},\ldots ,x_{T}\right)}^{T}$ be the vector of parameters describing the pose of each node $x_{i}$. Let ${\hat{z}}_{i}\left(x_{i}\right)$ and ${\hat{z}}_{ij}\left(x_{i},x_{j}\right)$ be the functions that compute the expected global and relative observations given the current estimation of the nodes. Following [33], for each unary and binary edge, we formulate an error function that computes the difference between the real and the expected observation:

(9) $$\begin{array}{cc} e(x_{i},z_{i}) & =e_{i}\left(x_{i}\right)=z_{i}-{\hat{z}}_{i}\left(x_{i}\right)\\ e(x_{i},x_{j},z_{ij}) & =e_{ij}\left(x_{i},x_{j}\right)=z_{ij}-{\hat{z}}_{ij}\left(x_{i},x_{j}\right) \end{array}$$

**Figure 10:**
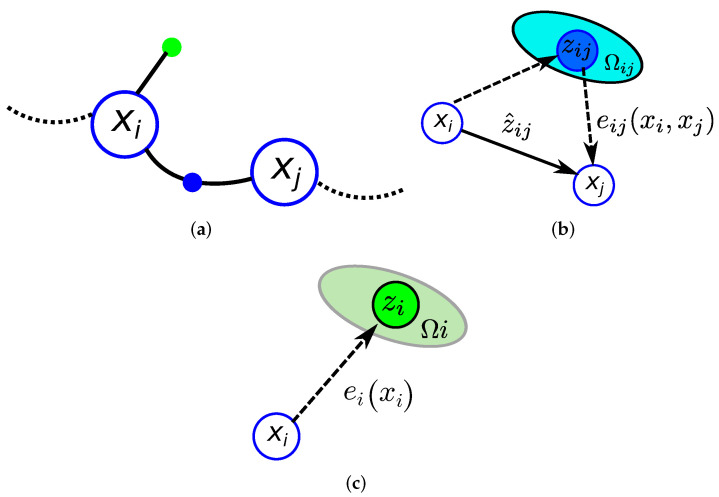
(**a**) Graphical representation of a portion of a pose graph where two nodes $x_{i}$ and $x_{j}$ are related by a binary edge (blue point) and where a unary edge is associated with node $x_{i}$ (green point). (**b**) Binary edge representing the relative position between the $x_{i}$ and $x_{j}$ nodes. (**c**) Unary edge corresponding to an absolute position associated with the $x_{i}$ node.

Figure 10 depicts a detail of the graphical representation used in this paper for the pose graph as well as the binary and unary edges.

The goal of a graph-based approach [33] is to determine the configuration of nodes $x^{*}$ that minimizes the sum of the errors introduced by the measurements, formulated as:

(10) $$x^{*}=\underset{x}{\mathrm{argmin}}\left(\sum_{i,j}e_{ij}^{T}\Omega_{ij}e_{ij}+\sum_{i}e_{i}^{T}\Omega_{i}e_{i}\right)$$

Equation (10) poses a non-linear least-squares problem as a weighted sum of squared errors, where $\Omega_{ij}$ and $\Omega_{i}$ are the information matrices of $e_{ij}$ and $e_{i}$, respectively. This equation can be solved iteratively using the Gauss–Newton algorithm.

Our approach for localization inside tunnels considers measurements coming from three sources of information (i.e., odometry data, gallery detection, and RF signal minima detection) and uses the procedures described in Section 5.1 and Section 5.2. Odometry measurements are straightforwardly introduced into the graph as binary constraints encoding the relative displacement between consecutive nodes $\left(x_{t-1},x_{t}\right)$. The output provided by the minima detection mechanism can be considered an absolute positioning system inside the tunnel that can be used as a unary constraint during the graph optimization process. Additionally, the absolute gallery location provided by the gallery detector algorithm is also introduced as a unary edge into the graph. Once the constraints derived from the measurements are incorporated into the graph, the error minimization process takes place, where the optimization time depends directly on the number of nodes.

Graph-based localization and mapping systems usually perform a rich discretization of the robot trajectory, where the separation between nodes ranges from a few centimeters to a few meters. This type of dense discretization would be intractable in a tunnel-like environment with few distinguishable features, where the length of the robot trajectory is measured in the order of kilometers. Therefore, it is necessary to maintain a greater distance between nodes to manage a sparser and more efficient graph.

These general criteria for introducing spread odometry nodes in the graph are modified when discrete features are detected. The following Section 6.1 and Section 6.2 describe the mechanisms used to incorporate the discrete measurements into the graph.

### 6.1. Management of RF Fadings Minima Detection in the Pose Graph

**Figure 11:**
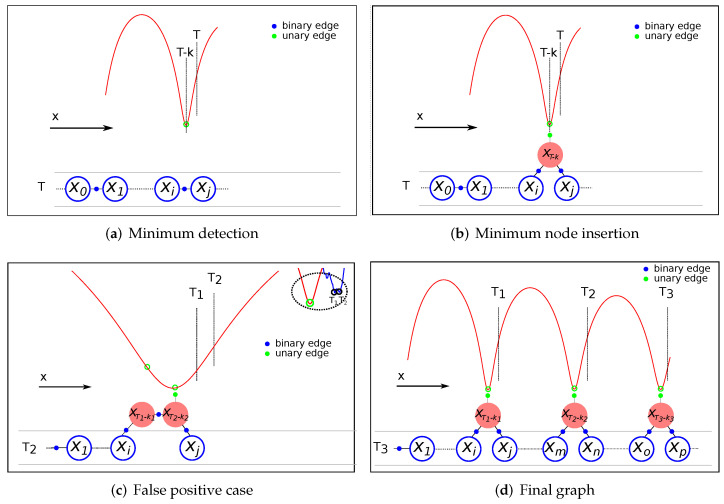
Pose-graph creation steps: (**a**) Minimum identification at time T. (**b**) Insertion of the node and the unary constraint corresponding to the detected minimum. (**c**) False positive case detail, deactivation of the previous unary edge. (**d**) Resulting pose-graph after three minimums.

As previously mentioned, RF signal minima detection is obtained at a posterior time *T* from when it occurred. This implies the need to incorporate information regarding an absolute position reference associated with a past robot position $x_{T-k}$ in the estimation process by means of a unary constraint. Due to the sparsity of the graph, the referred position is not likely represented in the graph by a previous node. Therefore, a mechanism to modify the current graph structure is needed to include the node corresponding to the point in the trajectory where the minimum was detected with its unary constraint. Figure 11 shows the procedure to introduce the unary measurement corresponding to a previous robot position $x_{T-k}$.

Suppose that an RF signal minimum corresponding to a previous time T−k is detected at timestamp *T*. Since the robot position $x_{T-k}$ is not present in the graph, the first step is to identify between which two nodes ($x_{i}$ and $x_{j}$) should be included based on the timestamps stored in each node and the odometry information corresponding to each timestamp (Figure 11a). The new node is then inserted into the graph connected to nodes $x_{i}$ and $x_{j}$ using their original relative odometry information. The unary edge with the reference position of the minimum is attached to the node $x_{T-k}$. Finally, to avoid double-counting information, the previous binary edge that relates $x_{i}$ and $x_{j}$ is deleted from the graph (Figure 11b). In the event of detecting another minimum corresponding to the same minimum in the RF map, the unary constraint of the previous minimum is deactivated, and the same procedure is followed (Figure 11c). Note that the node that initially corresponded to a minimum ($x_{T_{1}-k_{1}}$) is maintained in the graph as a regular node with the binary edge already created with the previous node, and the binary edge with the new minimum node. This can be the case for false positives or improved detections after the accumulation of more data.

Figure 11d presents an example of a final graph after detecting three RF minimums at different timestamps (i.e., T1, T2, and T3). As shown in this figure, the nodes (with their unary edges) are introduced into the graph representing their real positions corresponding to the minima occurrences, which highlights the simplicity of the pose graph approach to incorporating information from the past. Since the optimization process occurs after each graph modification, the estimation position is corrected by considering the newly incorporated restrictions.

### 6.2. Management of Gallery Detection in the Pose Graph

**Figure 12:**
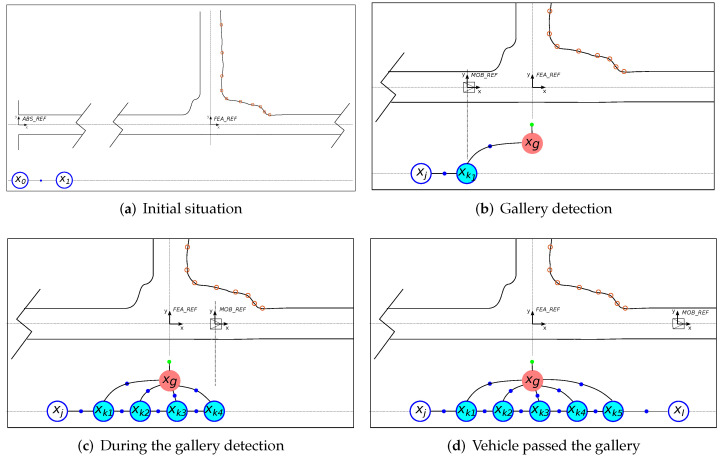
Gallery pose graph creation steps: (**a**) Initial situation. The vehicle starts moving. (**b**) The gallery is detected for the first time. (**c**) Nodes are added each time the gallery is detected. (**d**) Graph nodes once the vehicle has traversed the gallery. Blue dots denote binary edges and green dots denote unary edges. The robot is represented as traveling along the center of the tunnel and without heading variations to simplify the figure.

As previously stated, during the displacement of the robot along the tunnel, the discrete robot poses are represented by nodes in the graph. The relative odometry distance between them is encoded using binary edges, as shown in Figure 12a. In addition to the information introduced to the graph each time an RF minimum is detected, the graph is also enriched with information from the gallery detector, resulting in improved localization in the whole tunnel.

The gallery detector provides two different types of information: (a) the relative localization of the gallery and its associated error with respect to the robot positions from which the gallery is observed and (b) the absolute known position of the identified gallery. In this regard, we consider the gallery as a new feature of the environment whose position can be represented as a node in the graph. Then, the information provided by the gallery detector is introduced by using the types of edges that better suit their nature: binary edges encoding the relative observation between the robot and the gallery, and a unary edge associated with the gallery node encoding its a priori known absolute position.

The procedure for introducing the gallery information into the graph starts once a particular gallery is detected for the first time. Figure 12 illustrates this process, which consists of the following steps:

- At timestamp *T*, the uncertainty provided by the gallery detector complies with the criteria to consider that a gallery is detected. A new odometry node ($x_{k_{1}}$) corresponding to the robot position from which the gallery has been observed is added to the graph using the relative odometry information with respect to the previous node $x_{j}$.
- The first time the gallery is detected, the node corresponding to the gallery position ($x_{g}$) is also added to the graph using a binary edge that represents the relative observed position ($d_{k_{1}g}$) between the gallery and the current position of the robot. A unary edge encoding the absolute position of the gallery ($z_{g}$) is also attached to the gallery node. This gallery position is provided by the metric-topological map ($x_{g}^{A}$) (Figure 12b).
- Each time the gallery is detected, a new node ($x_{k_{i}}$) is inserted into the graph connected to the previous node using the relative odometry position and connected to the gallery node through a binary edge using the information provided by the gallery detector (relative distance $d_{k_{i}g}$ and uncertainty of the detection $\sigma_{k_{i}g}$). Both edges are encoded as binary edges (Figure 12c).
- When the gallery is no longer detected (i.e., the uncertainty raises above the threshold), the nodes are again introduced to the graph following the regular criteria (sparse graph) (Figure 12d).

It is worth noticing that the procedure to introduce the measurements from the gallery detector differs from the one proposed for the minima detector. Although the global positions for both types of features are known, their detection is produced in a different manner. As mentioned, in the case of the galleries, there is a relative observation that is instantaneous, so that this measurement and its associated uncertainty can be introduced into the graph at the time of its detection as a binary edge. However, in the case of the minima, the detection returns the value of the global position of the minimum, which is not instantaneous due to the necessity to accumulate signal readings to form the geometric shape of the minimum. This makes it more appropriate to represent that information as a unary edge associated with the past position from which the pattern of the minimum is centered.

To sum up, the pose graph is updated with the positions from which the gallery is observed, adding binary edges encoding relative distance constraints between nodes and a unary edge encoding an absolute position reference associated to the gallery node. As a result of this process, the localization resolution increases during the gallery detection. Again, the estimated position is corrected after executing the optimization process with each new node incorporation.

## 7. Experimental Results

To validate the proposed graph-based localization approach, all of the algorithms involved in the entire process were implemented in $MATLAB^{TM}$ and tested with real data collected during an experimental campaign carried out in the real-world scenario described in Section 3. The vehicle started from gallery number 17 (50 m from the Spanish tunnel entrance) and was driven up to gallery 6, traveling approximately 5000 m along the tunnel.

To the best of our knowledge, there are no publicly available datasets in these tunnel-like environments that we could use for comparison purposes. Moreover, few experimental results have been published in this field. The most relevant ones rely on the use of a UWB location system (see [31,34]), i.e., a GNSS-like approach for tunnels. The UWB localization systems obtain errors below 20 cm in tunnels but it is necessary to install an anchor device each 20 m inside the tunnel and to locate them accurately. Moreover, the experiments presented in the referenced works were conducted in a 30-m long gallery. Such a UWB-based localization system would require the installation of 250 anchor devices for the 5000 m tunnel length covered in the present work, which in most cases would be unfeasible.

Therefore, the objective of the experiments is to prove that our approach works in real environments. To highlight the effect of the discrete features detection on the cumulative localization error, we have not used the actual odometry, but a degraded one. The optimized instantaneous pose error depends on the quality of the odometry or enhanced odometry (possibly with scan matching or vision) used in continuous localization. However, as this section will show, this error will be reset to a maximum of 20 cm after each gallery detection and between 0.5 and 1.5 m after detection of RF signal minima.

In the experiments presented in this section, the robot navigated in a straight line along the center of the tunnel with negligible heading variations. This behavior during the experiment makes the simplification of the general formulation of our graph-based localization approach feasible, where x refers to (x,y,θ), a one-dimensional problem where *x* corresponds to the longitudinal distance from the tunnel entrance. The *y* and θ values are computed as described in Section 4.1. Hereafter, the magnitudes will be represented without bold type to be consistent with this simplification. During the displacement of the vehicle, the data provided by the sensors were streaming and logging with a laptop running a Robot Operating System (ROS) [35] on Ubuntu. For the validation process, the scan pattern-based gallery detector has been selected; however, both methods could have been used since they provide the same information for incorporation into the pose graph.

The real localization of the vehicle, which will be used as ground truth, is obtained by fusing all of the sensor data using the algorithm described in [36] with a previously built map. It is only feasible to apply this approach due to a very specific characteristic of the Somport testbed, which is that emergency shelters are placed every 25 m and thus serve as landmarks. It is noteworthy that the ground truth is only used for comparison purposes. The existence of these shelters is omitted by the proposed localization system to generalize the applicability of the method to other types of tunnels that do not include these characteristics.

Table 1 presents the absolute positions of each gallery $x_{g}^{A}$, as provided by the metric-topological map. These absolute positions, considered as the ground truth, correspond to reference points from which the gallery patterns were generated.

The position references of the minima $x_{m}^{A}$ extracted from the RF map are shown in Table 2. It should be noted that the periodicity of the RF signal under the defined transmitter-receiver setup is observable once the near sector is crossed (i.e., at a significant distance from the tunnel entrance). Therefore, during the route of the platform, there will be areas where the graph-based approach will only incorporate information from the galleries. However, there will be other areas where both the galleries and the RF signal minima coexist. Thus, the data provided by the detection of both features will be introduced into the graph.

### 7.1. Algorithm Implementation

As previously mentioned, the nodes are added to the graph at regular intervals corresponding to the distance traveled by the platform. The selected interval is 40 m, which is adequate to fulfill a twofold purpose: (a) providing sufficient discretization of the total distance traveled (in the range of km) while avoiding the complexity of a more dense graph and (b) ensuring sufficient resolution between tunnel feature detections. The constraints between two consecutive nodes ($x_{i},x_{j}$) are modeled with *binary* edges $\langle z_{ij},\Omega_{ij}\rangle$ using the relative position between them provided by the odometry as the measurement, $z_{ij}=\left(x_{j}^{odom}-x_{i}^{odom}\right)$ and the odometry uncertainty $\sigma_{odom_{ij}}$ for the information matrix $\Omega_{ij}={\left[\sigma_{odom_{ij}}^{2}\right]}^{-1}$. The uncertainty is scaled by the traveled distance from node *i* to node *j*. Table 3 shows the equations involved the first time a new regular node $x_{j}$ is introduced into the graph with the labeling of the binary edge. The expression used to compute the error at each iteration is also detailed.

The two detection processes run concurrently in real-time, waiting for the occurrence of a minimum or gallery event. When a minimum is detected at time *T*, a new node $x_{m}$ is introduced into the graph using the mechanism described in Section 6.1. First, the odometry robot position corresponding to the minimum occurrence ($x_{m}^{odom}$) is used to create *binary* edges with $x_{i}$ and $x_{j}$, as previously described. The position reference of the minimum $\left(x_{m}^{A}\right)$ provided by the RF map is considered as the measurement $z_{m}$ and is included as global information with a *unary* edge associated with this new minimum node, being $\Omega_{m}={\left[\sigma_{m}^{2}\right]}^{-1}$ (i.e., the information matrix). $\sigma_{m}$ corresponds to the uncertainty of the measurement provided by the RF map. Table 4 summarizes the process when a new minimum node is introduced into the graph for the first time with the unary edge and the error computed at each iteration.

Similarly, when a gallery is detected for the first time, a new node $x_{k_{1}}$ corresponding to the estimated robot position from which the gallery is observed is added to the graph using the odometry position ($x_{k_{1}}^{odom}$). A *binary* edge $\langle z_{jk_{1}},\Omega_{jk_{1}}\rangle$ is created between the previous node *j* and the new one using the same mechanism previously described in Table 3. At the same timestamp, a node representing the position of the gallery $x_{g}$ is also incorporated into the graph. The constraint between the $x_{k_{1}}$ and the gallery node $x_{g}$ is modeled by a *binary* edge $\langle z_{k_{1}g},\Omega_{k_{1}g}\rangle$ using the information provided by the gallery detector: the relative distance $d_{k_{1}g}$ between them as the measurement $z_{k_{1}g}$ and the detection uncertainty ($\sigma_{k_{1}g}$) for the information matrix $\Omega_{k_{1}g}$. Lastly, the absolute position of the gallery in the tunnel ($x_{g}^{A}$) is introduced into the graph as a global measurement $z_{g}$ by means of a *unary* edge $\langle z_{g},\Omega_{g}\rangle$ associated with the gallery node. Since the gallery global position is obtained from the geometrical map of the tunnel, it is considered a ground truth with a very low value for the uncertainty of the unary edge ($\sigma_{g}=10^{-4}$). Each time the gallery is detected from a new position, a new node $x_{k_{i}}$ is incorporated into the graph by encoding the constraints with the previous node ($x_{k_{i-1}}$) and the gallery node ($x_{g}$) by means of two binary edges, as previously described. Once the gallery is no longer detected because the vehicle has passed through the gallery area, the next gallery pattern is loaded to await the next detection. Table 5 shows the equations of the nodes and edges involved when a gallery is detected for the first time, along with the corresponding formulas to compute the errors.

Each time a new node or measurement is added to the graph, the optimization process occurs. The goal of this process is to determine the assignment of poses to the nodes of the graph, which minimizes the sum of the errors introduced by the measurements. The larger the information matrix ($\Omega_{ij},\Omega_{i}$), the more the edge matters in the optimization. In our case, the measurements associated with the minimum and gallery nodes have the smallest uncertainty since they are observations from the maps. Therefore, they play the role of “anchors” in the pose graph. As previously mentioned, the optimization process has been implemented in $MATLAB^{TM}$ based on the g2o back-end implementation [37].

Our approach guarantees continuous robot localization by accumulating the odometry data to the last estimated robot position in the graph—even in those areas where the node separation in the graph is large (when neither a gallery nor an RF minimum is detected).

### 7.2. Results

#### 7.2.1. Minima Detection

**Figure 13:**
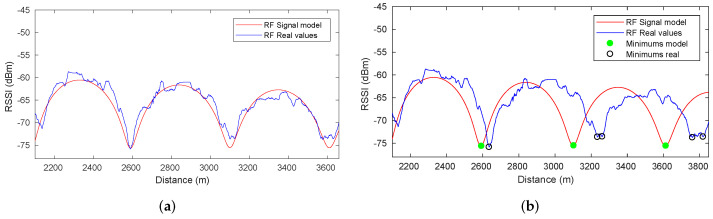
Results of the mimima detection process. (**a**) RF signal model and RF real values represented with respect to the ground truth. (**b**) RF real values with respect to the position estimated by the odometry without any correction due to the detection of previous discrete features.

Figure 13 presents the results of the minima detection method. The RSSI data provided by the RF receiver and the RF signal model are represented related to the ground truth in Figure 13a. As clearly shown, the RF sensor measurements in the real scenario are similar enough to the RF signal model to consider the latter as an RF map. The periodicity of the RF signal fadings under the transmitter-receiver setup of the Somport experiments was observed with sufficient signal quality from approximately 2.1- to 3.8-km points. As mentioned above, the original odometry was degraded to evaluate the strength of the detection systems. When the vehicle reached the area of the periodic fadings, some odometry error was accumulated as shown Figure 13b, where the RF real values are represented with respect to the position estimated by the odometry. Although the real signal waveform did not exactly match the RF signal model, the detection method can identify the real minima and associate them with the RF map minima, thereby providing the data required for incorporation into the graph. The error detection of the minima ranges from 0.5 to 1.5 m. The mechanism explained in Section 6.1 was used to handle this situation.

#### 7.2.2. Generic Gallery Detection

**Figure 14:**
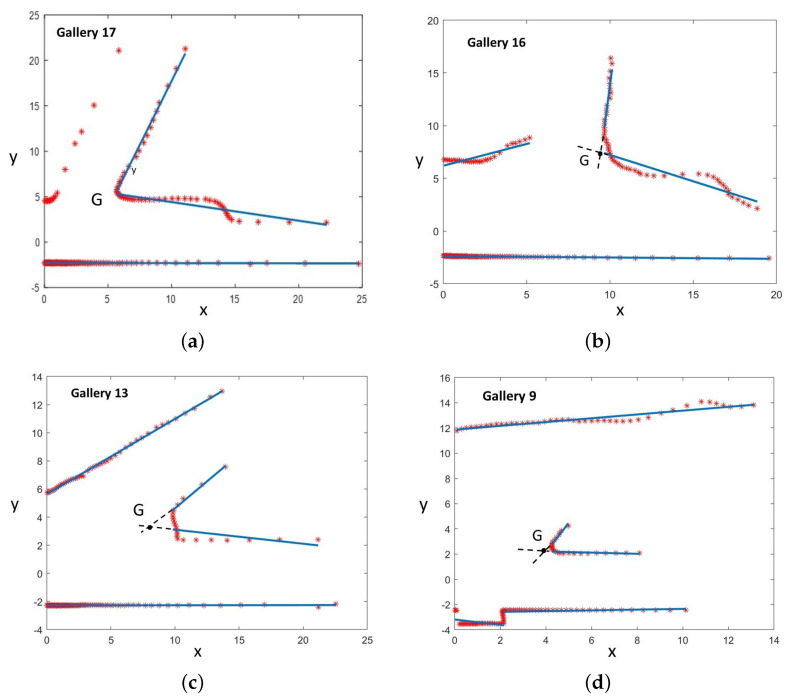
Four galleries recognized in the tunnel. Blue lines are the regression lines computed from the supporting points. The *G* point (i.e., the intersection of the extension of regression lines of the corner) is the location of the recognized gallery matching the gallery location in the topological map. (**a**–**d**) show galleries 16, 15, 13, and 9 from Table 1, respectively.

In Figure 14, four galleries with different shapes, inclinations, widths, dimensions, and supporting points were recognized by the generic gallery detection method. In this case, the intersection point in the corner corresponding to the representative point *G* associated with each gallery in the topological map was used to compute the gallery location in the global reference.

**Figure 15:**
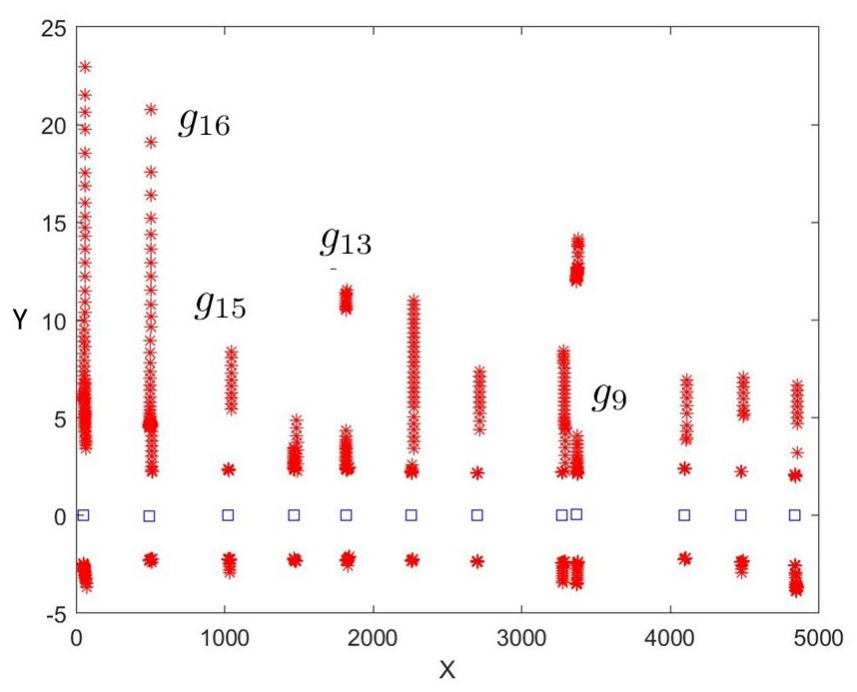
Squares represent the locations of the robot in front of every gallery. Each gallery is represented by laser points at the moment when the robot location was recomputed. The four galleries presented in Figure 14 are also shown.

A total of 12 galleries were detected. Neither false positives nor false negatives were detected. Their global locations were computed using Equation (7) and represented in Figure 15. The detector was able to recognize and distinguish them from other tunnel characteristics (e.g., lateral shelters, large holes on walls, small caves, etc.).

After applying Equation (8), the minimum, mean, and maximum values obtained for $\sigma_{x_{g}}$ along the tunnel were 0.01, 0.08, and 0.22 m, respectively. Only 16% of the estimated gallery locations have an uncertainty above 0.2 m.

#### 7.2.3. Pattern-Based Gallery Detection

**Figure 16:**
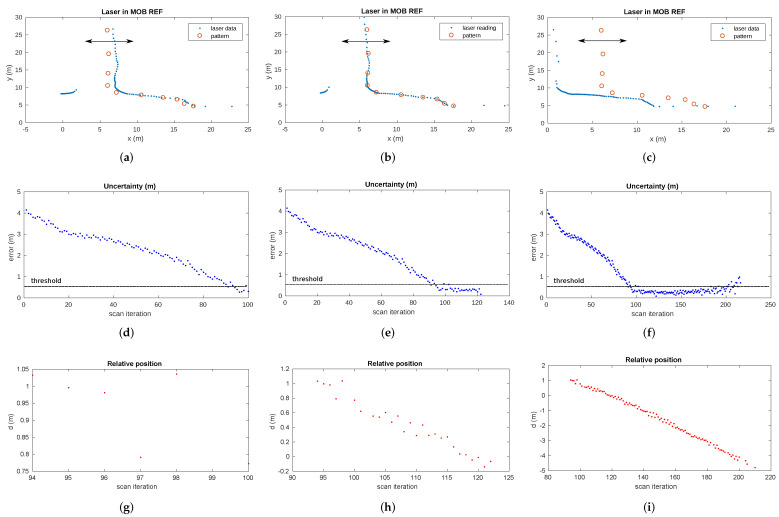
Gallery detection process. (**a**) First gallery detection timestamp. The detection uncertainty falls below the threshold (around the 90th iteration) (**d**) and the relative distance between the vehicle position and the gallery is provided by the detection algorithm (**g**). (**b**) The vehicle passes through the gallery position corresponding to the pattern reference. The uncertainty remains under the threshold (**e**) and the relative distance is provided during this time (**h**). (**c**) The vehicle (laser data) moves away from the gallery (pattern) and the uncertainty increases above the threshold (**f**). The condition of gallery detection is unsatisfied. (**i**) Relative distances between the vehicle position and the gallery during all the detection processes.

**Figure 17:**
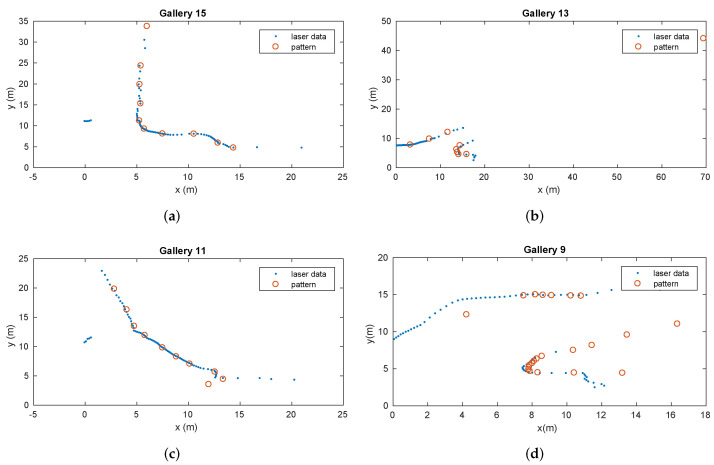
Matching process results for several galleries represented by different patterns during the vehicle displacement.

The results of the pattern-based gallery detector for gallery number 17 of the Somport tunnel are shown in Figure 16. The first row represents the laser scan reading along with the gallery 17 pattern at three different timestamps. The second and the third rows show the evolution of the uncertainty and the evolution of relative distance between the vehicle and the gallery, respectively. Figure 16a shows the first steps of the gallery observation from the vehicle. When the uncertainty $\sigma_{rg}$ falls below the threshold (Figure 16d), the gallery is considered detected and the relative distance $d_{rg_{17}}$ between the current position of the robot and the gallery is calculated (Figure 16g). The relative position of the gallery is provided by the detector as long as the uncertainty remains below the threshold (th), which is fixed at 0.5 m. Figure 16b,e,h presents the timestamp when the error in the scan-pattern matching process is close to 0. This situation corresponds to the vehicle passing by at the reference position of the gallery in the tunnel (FEA_REF). Lastly, when the uncertainty exceeds the threshold, the detection process of the current gallery is considered complete. Figure 16i presents the relative distance from the different vehicle positions to gallery 17 during the whole detection process. As previously stated, knowing the current estimated position of the vehicle and the movement direction, the pattern of the next expected gallery is loaded and the detection process starts again. Note that the new pattern is also loaded in case the gallery is not detected within the expected range of positions. The results of the matching process for other galleries are presented in Figure 17.

In the experiments in the Somport tunnel, uncertainty in the $d_{rg}$ estimation of less than 40 cm in 90% of the cases was assumed. However, when the robot was near the point where the pattern was acquired, the position error fell to a minimum. This corresponds to the point of less uncertainty in the robot position.

This minimum error had a mean value of 6.8 cm for the complete set of galleries. The best case was 1.9 cm for gallery 14 and the worst case was 12.7 cm for gallery 13.

**Figure 18:**
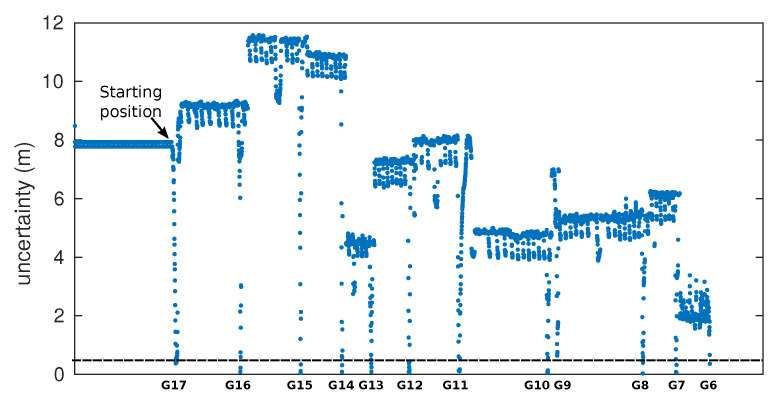
Evolution of the gallery detection uncertainty during the displacement of the vehicle from gallery 17 to gallery 6. The abrupt drops in uncertainty, indicating the detection of the gallery, have been previously depicted in detail in Figure 16d–f. The starting position of the vehicle corresponds to gallery 17.

The evolution of gallery detection uncertainty during the displacement of the vehicle along the tunnel can be observed in Figure 18. This figure clearly shows a sharp drop in uncertainty below the threshold, indicating the detection of each gallery.

#### 7.2.4. Graph-Based Localization Results

**Figure 19:**
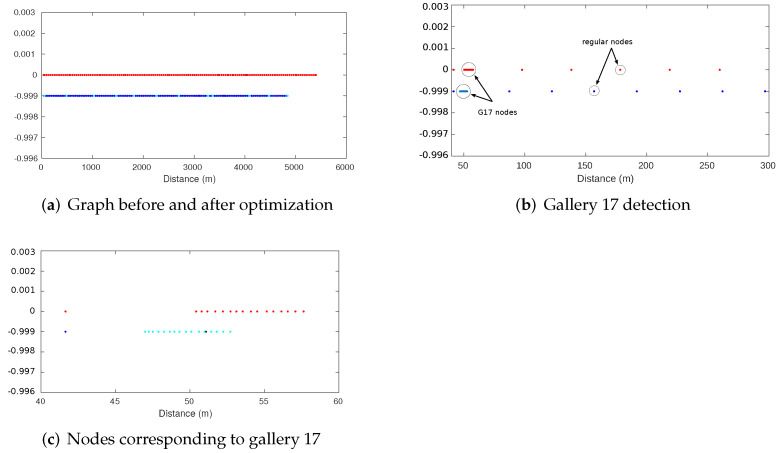
Node graph with RF minimums and gallery detections. (**a**) Complete node graph before (red) and after (blue) optimization. Due to the applied simplification to one-dimension problem, the y axis values have been set to those values only for visualization purposes, avoiding the overlapping of both graphs. (**b**) Area of the node graph showing the nodes incorporated during gallery 17 detection joined together with regular nodes before and after gallery detection. (**c**) Details corresponding to the nodes representing the vehicle position from which gallery 17 was detected (cyan). The node corresponding to the gallery position is shown in black.

The pose graph is continuously generated during the displacement of the vehicle along the Somport tunnel while incorporating information provided by odometry, RF minima detection, and gallery detection. Figure 19a shows the resulting graph with all sources of information before (red) and after optimization (blue). This figure highlights how the positions of the vehicle, represented by the nodes, have an obvious forward odometry drift corrected after the optimization. As previously mentioned, a sparse graph is maintained by the addition of trajectory nodes at long regular intervals. This criterion changes in the case of a gallery detection, with a node being added from each vehicle position from which the gallery is observed. This situation is represented in Figure 19b,c for gallery 17, where it is observed how the graph density increases in those areas, providing greater discretization. Similarly, when an RF minimum is detected, a node is incorporated at the position where the minimum occurred.

The resulting position graph corresponding to the entire displacement of the vehicle consists of 223 nodes and 306 edges. The total number of nodes includes 3 nodes representing the positions of the vehicle corresponding to RF minimum occurrences, 12 nodes representing the positions of the galleries present in the Somport tunnel, 81 nodes from which any of the galleries were detected, and 127 regular nodes. Table 6 summarizes these figures. The total quantity of edges includes unary edges encoding absolute positions related to minimums and gallery nodes (3 and 12, respectively) as well as binary edges, which represent relative constraints between nodes and gallery nodes (81) and between regular nodes (210). Table 7 presents the classification of edges.

**Figure 20:**
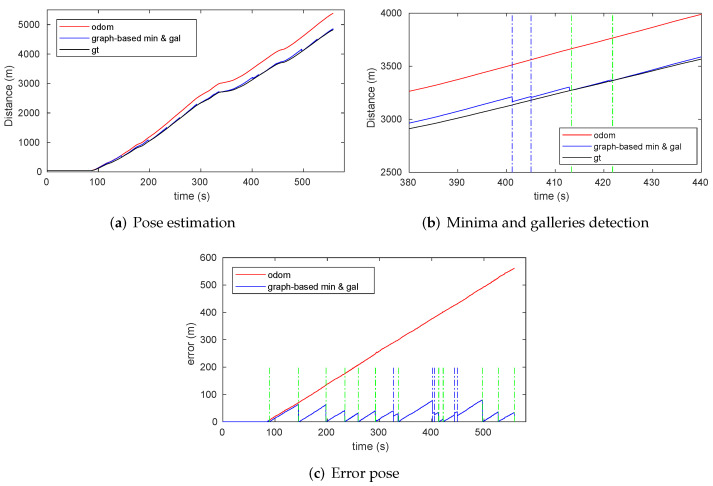
Results of the online pose graph localization approach. (**a**) Estimated position along the tunnel provided by the odometry (red) and our proposed approach (blue) in comparison with the ground truth (black). (**b**) Details of the estimated positions corresponding to the time slot when an RF minimum (dashed blue lines) and two galleries (dashed green lines) were detected. The position was corrected with each detection. (**c**) Position error during the displacement of the vehicle.

The results of our pose graph localization approach are represented in Figure 20. Figure 20a presents the pose estimation of the vehicle during the continuous localization process in comparison with the pose estimation using only odometry information. The position is corrected with each RF minimum or gallery detection, as shown in detail in Figure 20b. As a consequence of the optimization process, the localization error accumulated during the movement of the vehicle was reset each time a discrete feature providing an absolute position was detected, as shown in Figure 20c. It is worth noticing that, since the optimization process was applied during motion, the longitudinal error remained bounded every time a discrete feature was found, which also led to a decrease in the errors of previous instants. However, this is not reflected in Figure 20c since the graph represents the instantaneous error during the displacement of the vehicle and the representation of previous values was not modified.

**Figure 21:**
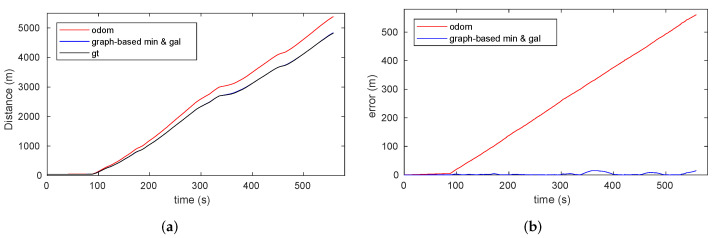
Results of the pose-graph approach after the service routine of the vehicle. (**a**) Pose estimation, (**b**) error pose.

One of the main benefits of the proposed approach is the ability to not only correct the error position online (during the displacement of the vehicle), but also to modify the location of certain features observed during the vehicle trajectory once the service routine is complete. Figure 21 presents the results for when the position and error are calculated once the tunnel has been traversed (i.e., when the optimization process occurs with all of the information included in the graph at the end of travel). Figure 21a presents how the estimated position obtained through our proposed method closely follows the true position of the vehicle, whereas the purely odometry-based estimation diverges from it. The position error along the tunnel remains bounded under very acceptable values in comparison with the resulting error from using only odometry information, which increases over time as shown in Figure 21b. As expected, higher position errors corresponded to areas where the distance between galleries is larger. It is worth noting that the position error was significantly flattened with respect to the online case, which demonstrates the strengths of this method for inspection applications.

### 7.3. Graph-Based Localization Performance Evaluation

To compare the performance of the graph-based localization approach using different sources of information, we used the metric proposed in [38]. The graph accuracy is not based on the calculation of absolute error among positions but rather on the creation of a graph consisting of virtual edges created by using the ground truth measurements evaluated in the estimated node positions. This well-known technique is commonly used to compare SLAM approaches that use different estimation techniques or different sensor modalities since all computations are made based on the corrected trajectory of the robot.

Table 8 presents a comparison of the overall mean $\chi^{2}$ error per edge during the localization process using different sources of information. Equation (11) shows the general expression used to calculate the $\chi^{2}$ value, where *n* is the number of edges.

(11) $$\chi^{2}=\frac{1}{n}\sum_{i,j}e_{ij}^{T}\Omega_{ij}e_{ij},\;\Omega_{ij}=I$$

As expected, using the most information sources during the localization process (i.e., minima and gallery positions as absolute measurements and odometry data as relative measurements) yielded the best result in terms of accuracy. When using only the galleries and the odometry data, the results were also good and better than those using only the minima and odometry. This is mainly because the galleries are distributed all along the tunnel, whereas the minima are only detected in a certain section. The greatest mean error was obtained when only odometry data were introduced into the graph.

Finally, regarding optimization times, the full graph converged in 0.23 ms after three iterations using the g2o framework running in an Intel Core i7 at 1.80 GHz processor.

## 8. Conclusions

In this paper, we have presented a graph-based localization approach for tunnel-like environments using different sources of information, including odometry data, absolute positions provided by an RF signal minima detector based on a theoretical fadings model acting as an RF map, and the absolute positions provided by a gallery detector.

Various strategies have been developed to incorporate the data provided by the proposed detectors into the graph. On the one hand, information from the past corresponding to the RF minima was introduced by revisiting the nodes stored in the graph and modifying the existing edges. On the other hand, the discretization of the graph was increased by adding the data of all robot positions at which the galleries were detected. In both cases, unary constraints were associated with the detected features—RF minima and galleries—using the position references obtained from the RF map and the metric-topological map.

The feasibility of the proposed approach has been validated with the data collected during an experimental campaign developed in a real tunnel scenario. The empirical results demonstrate the validity of all processes involved in the localization during the displacement of the vehicle.

In addition to the good performance of the detection methods demonstrated during the experiments, the results also indicate that the implemented approach allows the online correction of the localization error each time a new absolute measurement is added. This also leads to better localization during the detection of discrete features to avoid, for example, confusing similar galleries that are close to each other. The position error is further reduced if the optimization process is executed once the tunnel has been traversed (i.e., with all the information incorporated into the graph). As a result, it is possible to accurately locate features of interest observed during an inspection task in a service routine.

A performance evaluation of the graph was also developed, providing the expected results in terms of localization accuracy depending on the information sources used during the process.

Given the aforementioned information, we can state that our approach has several advantages over other methods addressed in the literature (typically probabilistic methods based on filters) to solve the localization problem in tunnels:

- It facilitates the easy incorporation of different sources of information. In the tunnel case, our method takes advantage of the features present in this type of environment (i.e., structural features in the case of galleries and periodic RF signal fadings).
- It allows us to keep track of the history of robot positions and revert from incorrect decisions produced during the estimation process (e.g., the integration of incorrect measurements). Therefore, it is also possible to incorporate information from the past that influences the present.
- This solution can be extrapolated to other types of environments of this type (e.g., pipes, sewers, and mines) by selecting the most appropriate sources in each case (e.g., the results of a scan matching process in the case of a more texturized environment).

The addition of more discrete features from the RF signal can easily be accomplished by adding a second RF receiver at a specific location on the platform, taking into account the effect of the cross-section antenna position over the fading structure. With this configuration, there is a 180-degree phase difference between signals, as shown in Figure 3b, which leads to a doubling of the RF minima and thus a resolution improvement for the proposed method. One limitation to be addressed in the future is related to the fast fadings area described in Section 2.3. The RF fadings periodicity is only observable at a significant distance from the RF transmitter once the higher modes are attenuated. One potential solution would be to add a second RF transmitter in the other extreme of the tunnel, provoking periodic fadings in the fast fadings area corresponding to the first RF transmitter. This improvement will be accomplished in future implementations. The integration of the proposed localization approach into a completely autonomous navigation system in a real prototype is also contemplated as future work in the field of inspection tasks in tunnels.

## Figures and Tables

**Table 1:** Galleries.

Gallery #	$x_{g}^{A}$ (m)	Gallery #	$x_{g}^{A}$ (m)	Gallery #	$x_{g}^{A}$ (m)
17	51	13	1813.9	9	3365.2
16	489.5	12	2260	8	4091.5
15	1026.9	11	2702.4	7	4475.8
14	1469.2	10	3275.1	6	4841

**Table 2:** RF minimums.

MIN #	$x_{m}^{A}$ (m)
1	2593.4
2	3103.6
3	3613.5

**Table 3:** First time a new node is introduced into the graph based on odometry information. The error is computed at each iteration.

Graph	Node	Binary Edge	Error
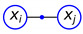	$x_{j}=x_{i}+d_{ij}^{odom}$ $d_{ij}^{odom}=x_{j}^{odom}-x_{i}^{odom}$	$z_{ij}=d_{ij}^{odom}$ $\Omega_{ij}={\left[\sigma_{odom_{ij}}^{2}\right]}^{-1}$ $\sigma_{odom_{ij}}=f\left(d_{ij}^{odom}\right)$	$e_{ij}=d_{ij}^{odom}-\left(x_{j}-x_{i}\right)$

**Table 4:** First time a new minimum node is introduced into the graph.

Graph	Node	Unary Edge	Error
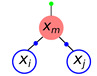	$x_{m}=x_{i}+d_{im}^{odom}$ $d_{im}^{odom}=x_{m}^{odom}-x_{i}^{odom}$	$z_{m}=x_{m}^{A}$ $\Omega_{m}={\left[\sigma_{m}^{2}\right]}^{-1}$	$e_{m}=x_{m}^{A}-x_{m}$

**Table 5:** First time a new gallery node is introduced into the graph.

Graph	Node	*Binary* Edge	*Unary* Edge	Error
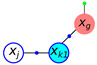	$x_{g}=x_{k_{1}}+d_{k_{1}g}$	$z_{k_{1}g}=d_{k_{1}g}$ $\Omega_{k_{1}g}={\left[\sigma_{k_{1}g}^{2}\right]}^{-1}$	$z_{g}=x_{g}^{A}$ $\Omega_{g}={\left[\sigma_{g}^{2}\right]}^{-1}$	$e_{k_{1}g}=d_{k_{1}g}-\left(x_{g}-x_{k_{1}}\right)$ $e_{g}=x_{g}^{A}-x_{g}$

**Table 6:** Resulting pose-graph nodes.

Nodes	Qty.
Minimum nodes	3
Gallery nodes	12
Nodes with gallery detection	81
Sparse regular nodes	127
Total	223

**Table 7:** Resulting pose-graph edges.

Edges	Qty.
Unary edges associated to minimum nodes	3
Unary edges associated to gallery nodes	12
Binary edges from nodes to galleries	81
Binary edges between sparse regular nodes	210
Total	306

**Table 8:** Graph accuracy analysis.

Source of Data	$\chi^{2}$
Only odom	12.87
Minima and odom	4.20
Galleries and odom	1.23
Minima, galleries and odom	1.15

## Data Availability

Not applicable.
